# Diaqua­bis(8-chloro-1,3-dimethyl-2,6-dioxo-1,2,3,6-tetra­hydro-7*H*-purinato-κ*N* ^7^)copper(II) dihydrate

**DOI:** 10.1107/S160053680802549X

**Published:** 2008-08-13

**Authors:** Ji-Hua Deng, Zhi-Xing Xiong, Yan-Ping Yi, Lin Yuan, Hui-Rui Guo, Meng-Ping Guo, Lin Liu

**Affiliations:** a College of Chemistry and Bio-engineering, Yichun University, Yichun, Jiangxi 336000, People’s Republic of China

## Abstract

The title mononuclear copper(II) complex, [Cu(C_7_H_6_ClN_4_O_2_)_2_(H_2_O)_2_]·2H_2_O, based on 8-chloro­theophylline (HCt), has the Cu atom at a center of symmetry in a slightly distorted *trans* square-planar geometry coordinated by two N atoms of two deprotonated HCt ligands and two O atoms of water mol­ecules. The crystal packing is stabilized by hydrogen bonds involving deprotonated HCt ligands, coordinated water mol­ecules and uncoordinated solvent water mol­ecules.

## Related literature

For related literature, see: Halpert *et al.* (2002 ▶); Antholine *et al.* (1985 ▶); García-Tojal *et al.* (1996 ▶); Okabe *et al.* (1993 ▶); Saryan *et al.* (1979 ▶); Serafin (1996 ▶); Spealman (1988 ▶); West *et al.* (1993 ▶); Zhao *et al.* (2003 ▶).
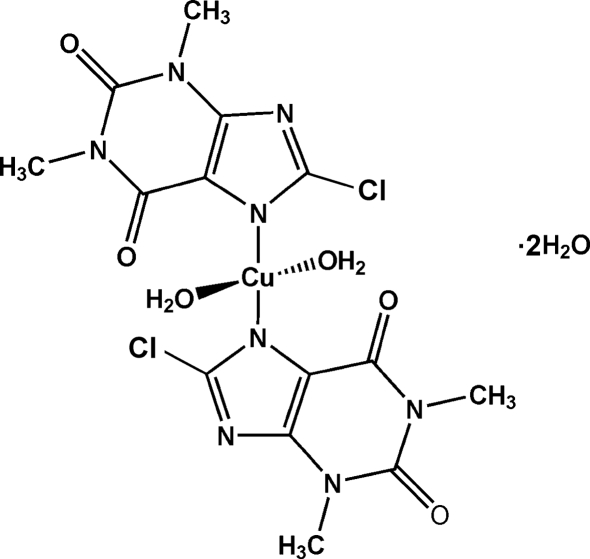


## Experimental

### 

#### Crystal data

[Cu(C_7_H_6_ClN_4_O_2_)_2_(H_2_O)_2_]·2H_2_O
*M* *_r_* = 562.82
Triclinic, 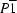

*a* = 8.377 (5) Å
*b* = 8.533 (8) Å
*c* = 8.830 (3) Å
α = 67.999 (2)°
β = 64.180 (7)°
γ = 78.388 (6)°
*V* = 526.2 (6) Å^3^
*Z* = 1
Mo *K*α radiation
μ = 1.35 mm^−1^
*T* = 293 (2) K
0.36 × 0.24 × 0.16 mm

#### Data collection

Bruker SMART CCD area-detector diffractometer
Absorption correction: multi-scan (*SADABS*; Sheldrick, 1996 ▶) *T*_min_ = 0.685, *T*_max_ = 0.802
3811 measured reflections
1834 independent reflections
936 reflections with *I* > 2σ(*I*)
*R*_int_ = 0.099

#### Refinement

*R*[*F* ^2^ > 2σ(*F* ^2^)] = 0.058
*wR*(*F* ^2^) = 0.102
*S* = 0.99
1834 reflections
153 parameters
19 restraints
H-atom parameters constrained
Δρ_max_ = 0.54 e Å^−3^
Δρ_min_ = −0.66 e Å^−3^

### 

Data collection: *SMART* (Bruker, 2004 ▶); cell refinement: *SAINT* (Bruker, 2004 ▶); data reduction: *SAINT*; program(s) used to solve structure: *SHELXS97* (Sheldrick, 2008 ▶); program(s) used to refine structure: *SHELXL97* (Sheldrick, 2008 ▶); molecular graphics: *SHELXTL* (Sheldrick, 2008 ▶); software used to prepare material for publication: *SHELXTL* (Sheldrick, 2008 ▶).

## Supplementary Material

Crystal structure: contains datablocks I, global. DOI: 10.1107/S160053680802549X/bg2201sup1.cif

Structure factors: contains datablocks I. DOI: 10.1107/S160053680802549X/bg2201Isup2.hkl

Additional supplementary materials:  crystallographic information; 3D view; checkCIF report

## Figures and Tables

**Table:** 

Cu1—O3	1.934 (5)
Cu1—N1	1.986 (6)

**Table:** 

O3^i^—Cu1—O3	180
O3—Cu1—N1	89.5 (2)
O3—Cu1—N1^i^	90.5 (2)
N1—Cu1—N1^i^	180

Symmetry code: (i) 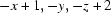.

**Table 2:** Hydrogen-bond geometry (Å, °)

*D*—H⋯*A*	*D*—H	H⋯*A*	*D*⋯*A*	*D*—H⋯*A*
O3—H3*A*⋯O2^ii^	0.82	1.98	2.729 (7)	154
O3—H3*B*⋯O4^iii^	0.84	1.81	2.612 (8)	159
O4—H4*A*⋯O1^iv^	0.82	2.07	2.897 (9)	176
O4—H4*B*⋯N4	0.82	2.03	2.839 (8)	170

Symmetry codes: (ii) 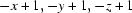; (iii) 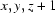; (iv) 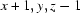.

**Fig. 1.**
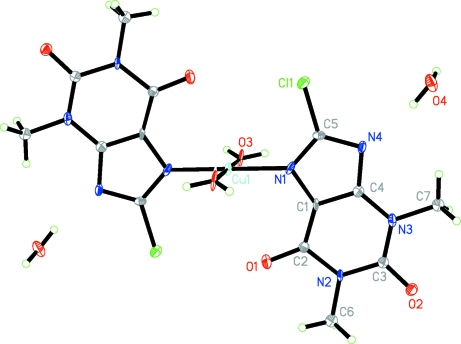
The structure of (I), showing 30% probability displacement ellipsoids and the atom-labeling scheme.

**Fig. 2.**
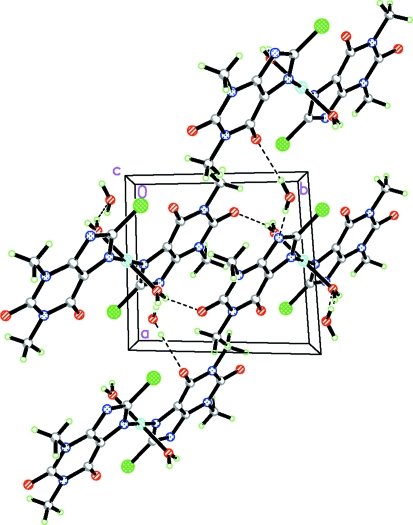
The crystal packing of (I).

## supplementary crystallographic information

### Comment

8-Chlorotheophylline (Ct) is a methylxathine drug related to caffeine and theophylline (Halpert *et al.*, 2002). It produces a number of effects, including nervousness, restlessness, insomnia, convulsions, anxiety, headaches and nausea (Serafin, 1996). The behavioural effects of this agent are attributed primarily to its ability to block adenosine receptors (Spealman, 1988). In recent years, many copper(II) complexes have draw attention due to the fact that they exhibit a greater biological activity, (antitumour, antibacterial, etc.)  than the corresponding free ligand because of their chelating ability and positive redox potential (García-Tojal *et al.*, 1996; West *et al.*, 1993; Antholine *et al.*, 1985; Saryan *et al.*, 1979).  Here, we report the structure of the title compound, {[Cu(Ct)_2_(H_2_O)_2_](H_2_O)_2_} (I), to our knowledge the first reported metal complex with 8-chlorotheophylline..

The stucture of (I) is shown in Fig. 1. It is composed of a mononuclear entity [Cu(Ct)_2_(H_2_O)_2_], together with two crystal water molecules; the copper^II^ atom, lying in a center of symmetry, is bonded to the nitrogen atoms of two individual 8-Ct molecules and oxygen atoms from two water molecules (Table 1), forming a *trans* square-planar arrangement. It should be noted that the ligand is in its anionic form (8-Ct^-^) in order to achieve charge balance.

Selected bond distances and bond angles are given in Table 1. The Cu—N and Cu—O bond lengths and bond angles at Cu1 are similar to those reported in some tetra-coordinated copper complexs (Zhao *et al.*, 2003; Okabe *et al.*, 1993). The 8-Ct molecule deviates slightly from planarity and the dihedral angle created by the least squares planes between the pyrimidine and imidazole ring is 1.2 (1) °.

The structure presents  O–H···O, O–H···N intermolecular hydrogen bonds (Table 2). between 8-Cts and water molecules. The coordinated water molecule is a donor towards the pyrimidine  O2 and the uncoordinated water O4, thus linking the complex units into a 2-dimentional structure along the *b* axis. Besides, the lattice water molecules acts as a donor towards the pyrimidine O1 and imidazole N4. These two hydrogen bonds serve to link the 2-D structures into a 3-D array along the *c* axis.

### Experimental

A solution of Cu(OAc)_2_.H_2_O (0.5 mmol) in water (5 ml) was slowly added to a solution of the ligand (1 mmol) in ethanol (14 ml) under stirring at room temperature. The mixture was sealed in a 25 ml Teflon-lined stainless steel vessel and heated under autogenous pressure at 383 K for 6 days, and then slowly cooled to room temperature. The green crystals obtained were recovered by filtration, washed with ethanol and dried in air. Yield: 52% (based on Cu).

### Refinement

Hydrogen atoms attached to carbon atoms were positioned geometrically and treated as riding, with C—H = 0.96 Å and *U*_iso_(H) = 1.2*U*_eq_(C). The water H atoms were located in a difference Fourier map, and were refined with a distance restraint of O—H = 0.82—0.84 Å and *U*_iso_(H) = 1.5*U*_eq_(O). The crystals are unstable outside the parental solution, for what the quality of the diffraction data was poor. This led to  unrealistic displacement parameters for four C and one O atoms, which were accordingly restrained to be nearly isotropic.

### Crystal data

**Table:**

[Cu(C_7_H_6_Cl_1_N_4_O_2_)_2_(H_2_O_1_)_2_]·2H_2_O	*Z* = 1
*M**_r_* = 562.82	*F*_000_ = 287
Triclinic, *P*1	*D*_x_ = 1.776 Mg m^−^^3^
Hall symbol: -P 1	Mo *K*α radiation λ = 0.71073 Å
*a* = 8.377 (5) Å	Cell parameters from 822 reflections
*b* = 8.533 (8) Å	θ = 2.6–25.0º
*c* = 8.830 (3) Å	µ = 1.35 mm^−^^1^
α = 67.999 (2)º	*T* = 293 (2) K
β = 64.180 (7)º	Block, green
γ = 78.388 (6)º	0.36 × 0.24 × 0.16 mm
*V* = 526.2 (6) Å^3^	

### Data collection

**Table:**

Bruker SMART CCD area-detector diffractometer	1834 independent reflections
Radiation source: fine-focus sealed tube	936 reflections with *I* > 2σ(*I*)
Monochromator: graphite	*R*_int_ = 0.099
*T* = 293(2) K	θ_max_ = 25.0º
φ and ω scans	θ_min_ = 2.6º
Absorption correction: multi-scan(SADABS; Sheldrick, 1996)	*h* = −9→9
*T*_min_ = 0.685, *T*_max_ = 0.802	*k* = −8→10
3811 measured reflections	*l* = −10→10

### Refinement

**Table:**

Refinement on *F*^2^	Secondary atom site location: difference Fourier map
Least-squares matrix: full	Hydrogen site location: inferred from neighbouring sites
*R*[*F*^2^ > 2σ(*F*^2^)] = 0.058	H-atom parameters constrained
*wR*(*F*^2^) = 0.102	*w* = 1/[σ^2^(*F*_o_^2^)]
*S* = 0.99	(Δ/σ)_max_ = 0.004
1834 reflections	Δρ_max_ = 0.54 e Å^−^^3^
153 parameters	Δρ_min_ = −0.66 e Å^−^^3^
19 restraints	Extinction correction: none
Primary atom site location: structure-invariant direct methods	

### Special details

**Table:**

Geometry. All e.s.d.'s (except the e.s.d. in the dihedral angle between two l.s. planes) are estimated using the full covariance matrix. The cell e.s.d.'s are taken into account individually in the estimation of e.s.d.'s in distances, angles and torsion angles; correlations between e.s.d.'s in cell parameters are only used when they are defined by crystal symmetry. An approximate (isotropic) treatment of cell e.s.d.'s is used for estimating e.s.d.'s involving l.s. planes.
Refinement. Refinement of *F*^2^ against ALL reflections. The weighted *R*-factor *wR* and goodness of fit *S* are based on *F*^2^, conventional *R*-factors *R* are based on *F*, with *F* set to zero for negative *F*^2^. The threshold expression of *F*^2^ > σ(*F*^2^) is used only for calculating *R*-factors(gt) *etc*. and is not relevant to the choice of reflections for refinement. *R*-factors based on *F*^2^ are statistically about twice as large as those based on *F*, and *R*- factors based on ALL data will be even larger.

### Fractional atomic coordinates and isotropic or equivalent isotropic displacement parameters (Å^2^)

**Table:**

	*x*	*y*	*z*	*U*_iso_*/*U*_eq_	
Cu1	0.5000	0.0000	1.0000	0.0214 (5)	
N1	0.5200 (8)	0.0829 (8)	0.7503 (7)	0.0214 (17)	
N2	0.1993 (8)	0.4308 (7)	0.6697 (7)	0.0203 (16)	
N3	0.4180 (8)	0.3889 (8)	0.4053 (7)	0.0264 (18)	
N4	0.6338 (8)	0.1570 (8)	0.4472 (8)	0.0245 (18)	
O1	0.1930 (7)	0.2659 (6)	0.9464 (6)	0.0296 (15)	
O2	0.2040 (6)	0.6046 (6)	0.3965 (6)	0.0210 (13)	
O3	0.6720 (6)	0.1598 (6)	0.9341 (6)	0.0340 (15)	
H3A	0.7154	0.2029	0.8256	0.051*	
H3B	0.7376	0.1651	0.9815	0.051*	
O4	0.8607 (7)	0.0978 (6)	0.1220 (6)	0.0330 (16)	
H4A	0.9578	0.1413	0.0704	0.049*	
H4B	0.7868	0.1226	0.2097	0.049*	
Cl1	0.8042 (3)	−0.1040 (3)	0.6122 (3)	0.0308 (6)	
C1	0.4156 (10)	0.2195 (9)	0.6872 (9)	0.0171 (19)	
C2	0.2692 (10)	0.2944 (9)	0.7842 (10)	0.019 (2)	
C3	0.2719 (10)	0.4812 (9)	0.4827 (9)	0.0142 (18)	
C4	0.4889 (11)	0.2531 (10)	0.5113 (10)	0.022 (2)	
C5	0.6432 (10)	0.0543 (9)	0.6041 (10)	0.019 (2)	
C6	0.0408 (9)	0.5307 (9)	0.7496 (9)	0.021 (2)	
H6A	0.0759	0.6360	0.7389	0.032*	
H6B	−0.0203	0.4686	0.8731	0.032*	
H6C	−0.0367	0.5527	0.6890	0.032*	
C7	0.4918 (9)	0.4281 (9)	0.2102 (8)	0.020 (2)	
H7A	0.3980	0.4704	0.1685	0.030*	
H7B	0.5465	0.3273	0.1809	0.030*	
H7C	0.5788	0.5122	0.1544	0.030*	

### Atomic displacement parameters (Å^2^)

**Table:**

	*U*^11^	*U*^22^	*U*^33^	*U*^12^	*U*^13^	*U*^23^
Cu1	0.0267 (10)	0.0224 (10)	0.0140 (9)	−0.0032 (8)	−0.0113 (8)	0.0001 (7)
N1	0.019 (4)	0.027 (4)	0.017 (4)	−0.011 (3)	−0.010 (3)	0.001 (3)
N2	0.027 (4)	0.021 (4)	0.009 (3)	−0.004 (3)	−0.004 (3)	−0.003 (3)
N3	0.030 (5)	0.036 (5)	0.011 (4)	−0.006 (4)	−0.004 (3)	−0.008 (3)
N4	0.022 (4)	0.033 (4)	0.012 (4)	−0.006 (3)	−0.002 (3)	−0.003 (3)
O1	0.036 (4)	0.033 (4)	0.014 (3)	−0.006 (3)	−0.007 (3)	−0.003 (3)
O2	0.0205 (16)	0.0215 (16)	0.0201 (15)	0.0010 (9)	−0.0110 (10)	−0.0035 (10)
O3	0.052 (4)	0.038 (4)	0.013 (3)	−0.031 (3)	−0.015 (3)	0.008 (3)
O4	0.027 (4)	0.050 (4)	0.018 (3)	−0.005 (3)	−0.010 (3)	−0.004 (3)
Cl1	0.0279 (15)	0.0291 (15)	0.0273 (13)	0.0002 (11)	−0.0096 (11)	−0.0031 (11)
C1	0.027 (5)	0.014 (5)	0.009 (4)	0.002 (4)	−0.010 (4)	−0.002 (3)
C2	0.019 (2)	0.019 (2)	0.019 (2)	−0.0004 (10)	−0.0083 (12)	−0.0056 (11)
C3	0.014 (2)	0.014 (2)	0.014 (2)	0.0005 (10)	−0.0066 (12)	−0.0031 (11)
C4	0.022 (2)	0.022 (2)	0.022 (2)	−0.0005 (10)	−0.0092 (12)	−0.0064 (12)
C5	0.011 (5)	0.019 (5)	0.028 (5)	−0.002 (4)	−0.002 (4)	−0.014 (4)
C6	0.021 (2)	0.021 (2)	0.020 (2)	0.0002 (10)	−0.0092 (12)	−0.0055 (11)
C7	0.020 (2)	0.020 (2)	0.019 (2)	0.0004 (10)	−0.0088 (12)	−0.0048 (11)

### Geometric parameters (Å, °)

**Table:**

Cu1—O3^i^	1.934 (5)	O2—C3	1.244 (7)
Cu1—O3	1.934 (5)	O3—H3A	0.8200
Cu1—N1	1.986 (6)	O3—H3B	0.8388
Cu1—N1^i^	1.986 (6)	O4—H4A	0.8242
N1—C5	1.329 (8)	O4—H4B	0.8243
N1—C1	1.401 (8)	Cl1—C5	1.711 (7)
N2—C3	1.407 (8)	C1—C4	1.333 (9)
N2—C2	1.442 (8)	C1—C2	1.351 (9)
N2—C6	1.472 (8)	C6—H6A	0.9600
N3—C3	1.369 (8)	C6—H6B	0.9600
N3—C4	1.402 (8)	C6—H6C	0.9600
N3—C7	1.479 (7)	C7—H7A	0.9600
N4—C5	1.361 (8)	C7—H7B	0.9600
N4—C4	1.347 (9)	C7—H7C	0.9600
O1—C2	1.234 (8)		
			
O3^i^—Cu1—O3	180.0 (3)	O1—C2—N2	118.4 (7)
O3^i^—Cu1—N1	90.5 (2)	C1—C2—N2	110.6 (7)
O3—Cu1—N1	89.5 (2)	O2—C3—N3	123.4 (6)
O3^i^—Cu1—N1^i^	89.5 (2)	O2—C3—N2	120.1 (7)
O3—Cu1—N1^i^	90.5 (2)	N3—C3—N2	116.5 (6)
N1—Cu1—N1^i^	180.000 (1)	C1—C4—N4	116.5 (7)
C5—N1—C1	104.1 (6)	C1—C4—N3	119.0 (7)
C5—N1—Cu1	131.8 (5)	N4—C4—N3	124.4 (7)
C1—N1—Cu1	122.9 (5)	N1—C5—N4	116.4 (7)
C3—N2—C2	125.4 (6)	N1—C5—Cl1	122.0 (6)
C3—N2—C6	115.4 (6)	N4—C5—Cl1	121.6 (6)
C2—N2—C6	119.2 (6)	N2—C6—H6A	109.5
C3—N3—C4	120.1 (6)	N2—C6—H6B	109.5
C3—N3—C7	118.8 (6)	H6A—C6—H6B	109.5
C4—N3—C7	121.0 (6)	N2—C6—H6C	109.5
C5—N4—C4	98.7 (6)	H6A—C6—H6C	109.5
Cu1—O3—H3A	109.4	H6B—C6—H6C	109.5
Cu1—O3—H3B	132.7	N3—C7—H7A	109.5
H3A—O3—H3B	112.4	N3—C7—H7B	109.5
H4A—O4—H4B	118.2	H7A—C7—H7B	109.5
C4—C1—C2	128.2 (7)	N3—C7—H7C	109.5
C4—C1—N1	104.3 (7)	H7A—C7—H7C	109.5
C2—C1—N1	127.5 (7)	H7B—C7—H7C	109.5
O1—C2—C1	131.0 (7)		

Symmetry codes: (i) −*x*+1, −*y*, −*z*+2.

### Hydrogen-bond geometry (Å, °)

**Table:**

*D*—H···*A*	*D*—H	H···*A*	*D*···*A*	*D*—H···*A*
O3—H3A···O2^ii^	0.82	1.98	2.729 (7)	154
O3—H3B···O4^iii^	0.84	1.81	2.612 (8)	159
O4—H4A···O1^iv^	0.82	2.07	2.897 (9)	176
O4—H4B···N4	0.82	2.03	2.839 (8)	170

Symmetry codes: (ii) −*x*+1, −*y*+1, −*z*+1; (iii) *x*, *y*, *z*+1; (iv) *x*+1, *y*, *z*−1.
